# Use of a monitoring tool for growth and development in Brazilian children – systematic review

**DOI:** 10.1016/j.rppede.2015.12.002

**Published:** 2016

**Authors:** Ana Claudia de Almeida, Larissa da Costa Mendes, Izabela Rocha Sad, Eloane Gonçalves Ramos, Vânia Matos Fonseca, Maria Virginia Marques Peixoto

**Affiliations:** a Instituto Nacional de Saúde da Mulher, da Criança e do Adolescente Fernandes Figueira (IFF/Fiocruz), Rio de Janeiro, RJ, Brazil; b Organização Não Governamental Casa da Árvore, Rio de Janeiro, RJ, Brazil

**Keywords:** Children's health, Growth and development, Development, Child care

## Abstract

**Objective:**

To assess the use of a health monitoring tool in Brazilian children, with emphasis on the variables related to growth and development, which are crucial aspects of child health care.

**Data source:**

A systematic review of the literature was carried out in studies performed in Brazil, using the Cochrane Brazil, Lilacs, SciELO and Medline databases. The descriptors and keywords used were “growth and development”, “child development”, “child health record”, “child health handbook”, “health record and child” and “child handbook”, as well as the equivalent terms in Portuguese. Studies were screened by title and summary and those considered eligible were read in full.

**Data synthesis:**

Sixty-eight articles were identified and eight articles were included in the review, as they carried out a quantitative analysis of the filling out of information. Five studies assessed the completion of the Child's Health Record and three of the Child's Health Handbook. All articles concluded that the information was not properly recorded. Growth monitoring charts were rarely filled out, reaching 96.3% in the case of weight for age. The use of the BMI chart was not reported, despite the growing rates of childhood obesity. Only two studies reported the completion of development milestones and, in these, the milestones were recorded in approximately 20% of the verified tools.

**Conclusions:**

The results of the assessed articles disclosed underutilization of the tool and reflect low awareness by health professionals regarding the recording of information in the child's health monitoring document.

## Introduction

The function and use of a child health monitoring tool have been discussed in the context of primary health care policy over the past three decades in Brazil.^1^^–^ 5 This tool's form, features, and content have gone through many changes. Furthermore, it had its goals and target audience expanded in an attempt to become an effective tool in child health promotion.^3^^,^ 6 ^,^ 7

In those same three decades, the economic, social and demographic transformations have changed the epidemiological profile of the population.^8^^,^ 9 These were accompanied by changes in the country's policy and health system,^10^ which caused a reordering of priorities in the Brazilian public health agenda.^4^^,^ 5 There have been many advances in the indicators of primary care, such as increased access to prenatal and immunization services and breastfeeding rates, and all contributed to the decline in child mortality.^8^^,^ 11 All these changes have posed new challenges to ensure the health of a growing and developing individual.^12^^–^ 15 It also caused the transition from a model of care focused on acute illness to one based on the integration of health services and intersectoral health promotion.^8^^,^ 10 ^,^ 16

In this transition, the Family Health Program (FHP) is the key strategy to restructure the care model of the Brazilian Unified Health System (Sistema Único de Saúde—SUS) since 1994.^10^ The first contact of the population with the local health system is through the family health teams, which coordinate care and seek to integrate health services. The health promotion activities go beyond the walls of the health centers and take place in the territory, that is, in the homes and community,^10^ and it is in the performance of such activities that the child monitoring tool recovers its historical function.^17^

The actions carried out in child's primary health care are essential for early detection of potential growth and development changes, as well as to decrease morbidity and mortality risks. Child growth is a dynamic and continuous process of differentiation from conception to adulthood, which depends on the interaction of biological characteristics and life experiences in the environment.^2^^,^ 17 The best monitoring method is the periodic record of the child's weight and height^18^ and, currently, the body mass index (BMI).^5^ The development, in turn, is broad and refers to a progressive transformation that also includes growth, maturation, learning, and psychic and social aspects.^2^ Its monitoring involves activities that assess steps or milestones of psychomotor development of children in each age group and can detect problems and changes in child development.^19^

Originally, the Child Health Card (CHC), proposed for the country in 1984,^2^ was the monitoring of basic actions of the Ministry of Health (MOH) for child health. From 1984 to 2003,^2^^,^ 3 the CHC has been modified and revised, with the addition of children's rights and some milestones of child development. The adoption of the CHC was explicitly mentioned in 2004 in the Agenda of Commitments for Complete Health and Mortality Reduction.^4^

In 2005, the CHC has taken the form of a booklet and is now called the Child Health Record (CHR).^6^^,^ 7 In this booklet, new information has been added for families and healthcare professionals in order to expand knowledge in child care and facilitate the understanding of aspects related to their growth and development. CHR is considered by the MOH a key tool for monitoring the promotion activities of the child's full potential of growth and development and preventing prevalent childhood diseases. Currently, the MOH distributes three million copies of the CHR to the municipal departments, which must pass them to public and private hospitals. It is a free document delivered to the newborn's family. There is no quantitative study compiling evidence from previous studies regarding the use of CHC/CHR.^17^^,^ 20 ^–^ 26 Therefore, the purpose of this article is to perform a systematic review to assess the completeness of CHC or CHR by health professionals in Brazil, based on evidence published in the literature, with emphasis on variables of monitoring the growth and development of the child.

## Method

The search was performed without restriction on year of publication in the following electronic databases: Cochrane Brazil, Latin American and Caribbean Health Sciences (Lilacs), Scientific Electronic Library Online (SciELO), Medical Literature Analysis and Retrieval System Online (Medline) and reference lists of articles, according to Preferred Reporting Items for Systematic reviews and Meta-Analyses (Prisma).^27^ The following descriptors and keywords were used: “growth and development”, “child development”, “child health record”, “child health handbook”, “health record and child”, and “child handbook”.

The articles included attended the following criteria for methodological quality^28^: hypotheses or defined objectives, outcome description, characteristics of participants, studied variables, main results and characteristics of losses, and adequacy of statistical tests used.

This review includes only works performed in Brazil and published in indexed journals, which measured the use of the growth and development monitoring tool prepared and distributed by the Ministry of Health from 1984, and quantitatively assessed the filling out of booklets.

Exclusion criteria were review articles, manuals, and completion of course work; the method of data analysis was qualitative, restricted only to vaccination or those whose sample consisted of specific risk groups, such as low birth weight and prematurity, with genetic and underlying diseases.

The 1984 version of CHC is a brochure on coated paper, printed in different colors and sizes for boys and girls, which can be folded in three, with spaces for child identification data, consultations, weight measurement according to age, growth monitoring chart up to 5 years old, and immunizations done. Since 1995, CHC included 11 milestones of child development with spaces to record the age in which they were achieved.

CHR, in booklet format that has been reprinted since 2005, has spaces for recording information of the basic health care of children from gestation to 9 years old, complications, treatments and graphics to indicate the variation of weight-for-age, height, head circumference (HC), and BMI. It also provides a space for recording the presence of the psychomotor developmental milestones according to the child's age.

The CHR should be filled in the routine follow-up visits. The Ministry of Health recommends seven visits in the first 12 months (1st week and 1st, 2nd, 4th, 6th, 9th, and 12th month), two in the second year (18th and 24th month), and from that age on, one visit per year.^7^

## Results

**Figure 1:**
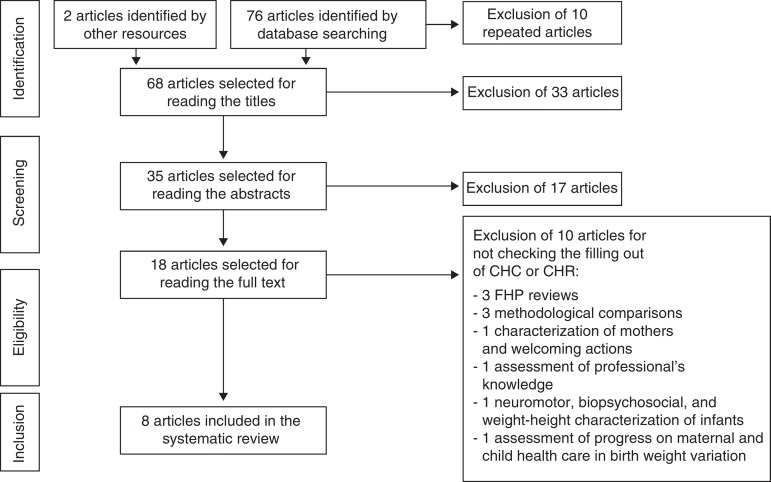
Identification flow, screening, eligibility, and inclusion of articles in the systematic review.

Sixty-eight non-repeated articles were identified in the electronic databases and reference lists (Fig. 1). In the first screening stage, four qualitative theses and 29 articles were excluded by reading the titles. Of these, 12 studies were restricted to vaccination, nine involve risk groups and/or underlying disease, three were of instructional materials (handbooks), three copies of booklets, one review, and one professional training study in primary health care (PHC).

In the second screening stage, 17 articles were excluded after reading the abstracts for not verifying the CHC/CHR completion. Eleven articles may be grouped as evaluation studies: three of nutritional indicators, three of Supervised Practical Activities, two of care practices, two of records analysis, and one of professionals’ knowledge. Five articles may be grouped as qualitative studies: two studies of the meaning of child care, one discourse analysis, one experience report, and one multidisciplinary approach to growth and development follow-up. In addition to these, a literature review of the role of nurses in children's nutritional health is excluded.

Eighteen articles remained for full text reading. Ten articles^29^^–^ 38 were excluded for not quantitatively assessing the children's health monitoring tools (Table 1). Of the eight included articles (Table 2), five evaluated the filling out of CHC^17^^,^ 20 ^–^ 23 and three^24^^–^ 26 of CHR. The searches were made in the Northeast,^17^^,^ 21 ^,^ 23 Southeast,^20^^,^ 24 ^,^ 25 South,^26^ and Midwest regions.^22^

**Table 1:** Articles selected for reading the full text and excluded from systematic review.

Title	Author (s)	Reason for exclusion
Evaluating child healthcare in the context of Family Healthcare in the city of Teixeiras, Minas Gerais (MG, Brazil)	Da Costa et al. 29	Classification used in the analysis for CHC filling out was “incomplete” without specifying frequencies for the variables of interest.
A case study of the Community Health Agents Program in Uruburetama, Ceará (Brazil)	Ávila 30	Monitoring actions of child growth and development were evaluated on the technical aspects and agents’ practices.
Nutritional evaluation of children aged from six to sixty months	Sousa and Araújo 31	Nutritional assessment of children was performed by comparing the criteria of Waterlow and weight/age curve of CHR.
The evolution of maternal and child healthcare and birth weight in the State of Pernambuco in 1997 and 2006	Noronha et al. 32	Information was collected from the child's guardian. Birth weight was collected from CHC when recorded.
Children health care evaluation (0–5 years) according to users’ perceptions in the Family Health Strategy of Teresópolis, Rio de Janeiro State	Ribeiro et al. 33	Linking with public institutions of child care was evidenced by the possession of CHC.
Monitoring of child growth: knowledge and practices of nurses in primary health care	Reichert et al. 34	Growth charts were used in verification issues of professionals’ knowledge.
Child development: agreement between the child health handbook and the guide for monitoring child development	Oliveira et al. 35	Classifications of development were compared according to the CHR and the Manual for Child Development Monitoring in the Context of IMCI.
Evaluation of childhood development: an interdisciplinary challenge	Alvim et al. 36	Classifications of development were compared according to the CHR and the Manual for Child Development Monitoring in the Context of IMCI.
Neuromotor, growth and biopsychosocial profile of latents	Rothstein and Beltrame 37	Child Health Card was used only as a source document information.
User embracement and maternal characteristics associated with liquid offer to infants	Niquini et al. 38	Mothers were asked if they have received the mother-infant welcoming card in the maternity. There was no analysis of filling out variables.

**Table 2:** Description of the studies included in the systematic review.

Authors	Objective	Data collection site	Conclusion
Santos et al. 20	Assess the primary care offered to the mother and child population.	Vaccination station, city of Teresópolis (RJ).	Despite being a child care visit, 30% of children did not have their weight recorded in the CHC.
Ratis and Batista 17	Evaluate the structure and process of growth monitoring.	Health units of PE State.	The lack of interest in growth monitoring was more prominent upstate.
Carvalho et al. 21	Evaluate the growth monitoring.	Health units of PE State.	Growth monitoring indicators do not greatly exceed 50%, and were lower upstate.
Sardinha and Pereira 22	Assess the filling out of CHC.	Health centers in the cities of the DF.	The weight chart filling was more accomplished in younger children.
Vieira et al. 23	Assess the filling out of CHC.	Health units of Feira de Santana (BA).	The filling out of the growth curve was complete in 41.1% (905) and development chart in 7.8% (170).
Goulart et al. 24	Assess the filling out of CHR and know the mothers’ perception about it.	UBS of Belo Horizonte (MG) and home visit. Santana (BA).	Birth weight was the field most filled out (91%). Failures suggest that CHR does not meet its goal.
Alves et al. 25	Assess the quality of CHR's filling.	UBS of Belo Horizonte (MG).	The best fill percentages were on the identification, vaccination record, and birth data.
Linhares et al. 26	Assess the filling out of CHR and know the mothers’ perception about it.	Home visits in areas of four UBS of Pelotas (RS).	The CHR's filling was limited to sections that were already present in the CHC.

Information was obtained from questionnaires addressed to the mother or child's guardian, or to the directors of health services, or was collected directly from the instrument studied. The surveys were made in services within the public health network and home visits.

The variability of the measured items and of the evaluation criteria of filling out the tool made it difficult to compare the filling frequency for all items of CHC or CHR.

The percentage of tools filled out with data regarding identification, pregnancy monitoring, and birth is presented in Table 3. In 2005, only 55.6% of the CHR had the name of the child filled in.^24^ The authors reported that the mean age of these “unnamed” children was 68 days (2.2 months), median of 59 days (1.9 months), time at which this information should have been filled out by health professionals after several opportunities to see child—in the maternity and primary care visits. We also noted that there was an increase in the percentage of CHC/CHR filled out between 2005 and 2008 for all the identification variables, except for the number of the Certification of Live Birth (CLB). The highest increase (four-folds) was in the number of Birth Certificates (BC).

**Table 3:** Filling out percentage of identification, pregnancy and birth monitoring data reported in the studies included in the systematic review. a

Authors		Ratis and Batista 17	Carvalho et al. 21	Vieira et al. 23	Goulart et al. 24	Alves et al. 25	Linhares et al. 26
*Research year*		1998	1998	2001	2005	2006	2008
*Document*		CHC	CHC	CHC	CHR	CHR	CHR
*N*		1194	662	2215	797	355	107
*Age*		<5 years	<12 months	≤12 months	<9 months	<16 months	<12 months

*Identification*							
	Name	–	–	99.8	55.6	93.8	93.5
	Birth date	–	–	99.3	90.1	99.7	100
	Birthplace	–	–	76.6	–	–	98.1
	Mother's name	–	–	–	90.7	98.9	99.1
	Address	–	–	–	38.9	–	73.8
	Telephone	–	–	–	22.1	–	47.7
	Neighborhood	–	–	–	33.4	–	67.3
	Zip code	–	–	–	14.6	–	21.5
	City	–	–	–	34.3	–	64.5
	Ethnicity/Color	–	–	–	50.1	–	66.4
	N° CLB	–	–	–	60.9	–	33.6
	N° BC	–	–	–	2.0	–	8.4

*Gestation*							
	Prenatal	–	–	–	59.6	58.0	–
	N° prenatal visit	–	–	–	68.5	69.9	–
	Serology	–	–	–	50.0	–	–
	Type of delivery	–	–	93.3	84.9	89.3	–
	Birth						
	Gestational age	–	–	–	75.8	72.4	–
	Apgar5′	–	–	28.4	76.7	53.5	–
	Weight	86.8	89.4	97.2	91.1	96.9	–
	Length	–	–	91.8	89.6	91.2	–
	Head circumference	–	–	88.9	84.9	85.6	–

a The studies by Santos et al. 20 and Sardinha and Pereira 22 showed no filling out results of identification, pregnancy monitoring, and birth.

CLB, Certificate of Live Birth; BC, Birth Certificate.

Only one study evaluated the serology data filled in during prenatal^24^ and found that this was the lowest filling percentage of the pregnancy monitoring variables: about 50% of the CHR studied.

Birth weight was the most described record among the variables related to the child's birth (Table 3). There was an increase in the filling percentages among the studied CHC/CHR, but there was a decrease when the tool changed, such as the gestational age, for example. Between 2001 and 2006, there was an increase in the filling out of Apgar and little variation in the filling of height and head circumference.

The results of the monitoring variables of growth and development are shown in Table 4. Only two studies^20^^,^ 26 reported consultation records concerning growth. The lowest percentage of CHR filling out was 74.6%, weight monitoring in 1998.^20^ However, 10 years later, the weight, height, and HC records were more than 80% filled out in the work by Linhares et al.^26^

**Table 4:** Filling out percentage of growth and development monitoring variables in the studies included in the systematic review *

Authors		Santos et al. 20	Ratis and Batista 17	Carvalho et al. 21	Sardinha and Pereira 22	Vieira et al. 23	Alves et al. 25	Linhares et al. 26
*Tool*		CHC	CHC	CHC	CHC	CHC	CHR	CHR
*Age*		<12 months	<5 years	<12 months	<5 years	≤12 months	<16 months	<12 months
*Growth*								
*Data from medical visits*								
		( *N* =299)						( *N* =107)
	Visit date							91.6
	Age							90.7
	Weight	74.6 a						89.7 b
	Height							87.9 b
	Head circumference							82.2 b

*Birth data in charts*								
			( *N* =1193)	( *N* =662)			( *N* =355)	
	Weight		36.9	44.1			69.3	
	Head circumference						15.5	

*Data from visits in charts*								
		( *N* =307)	( *N* =624)	( *N* =402)	( *N* =3543)	( *N* =2200)	( *N* =355)	( *N* =107)
	Weigh	70.4 a	59.9 c	58.2 d	21.1 e	41.1 f	59.4 g	96.3 h
	Length/height							42.1 h
	Head circumference						30.7 g	35.5 h
	Development							
						( *N* =2191)	( *N* =355)	
	Milestones 0–36 months					7.8 i	18.9 j	

* Goulart et al. 24 did not present filling out data on the variables of growth and development monitoring.

a At least one record in the three months prior to the interview.

b Records according to the child's age.

c Records in the consultation day.

d Last updated record.

e Records properly punctuated, according to the Ministry of Health.

f At least one record every three months.

g Weight and HC records marked on the chart whose difference between the age at the time of the record and the child's chronological age was ≤3 months.

h At least one record verified.

i All records matching the child's age.

j Records in three or more age groups present in CHR.

Records of weight and HC at birth in the graphs showed low frequency of CHR filled out. In works performed in Pernambuco, birth weight at birth was only indicated on the chart in 36.9%^17^ and 44.1%^21^ of the cards, although it was recorded in 86.8%^17^ and 89.4%^21^ (Table 4), respectively, of these cards. Similarly, in Belo Horizonte,^25^ only 69.3% and 15.5% of the CHR had markings on the charts of weight and HC at birth, respectively.

The filling out percentage of the weight-for-age chart showed great variation between studies (21.1–96.3%) due to the criteria used to consider the filling out as appropriate. For children up to one year, when a record every three months was required, Vieira et al.^23^ reported 41.1% of adequate filling out in the weight-for-age chart. In the study that considered a single marking as sufficient, a percentage of 96.3% was reported.^26^ In the Federal District,^22^ 21.1% of correct filling out were found, according to the recommended by the Ministry of Health. It was found that the filling out percentage decreased with age, from 53.8% in the age group up to five months to 6.6% in the age group of 48–60 months. In Pernambuco,^17^ 59.9% of CHC had a record in the weight chart on the day of consultation. In this same work, according to the child's age, 38% of CHC had none or only one weight record in the chart. The condition “no point recorded” is similarly distributed in all age groups: 27.8% (<12 months), 21.7% (12–24 months), and 27.2% (48–60 months). However, 40.5% of the CHC had two to six points on the chart. Of these, 46% in the age group were under one year and 29.7% between 48 and 60 months.

Linhares et al.^26^ were the only ones to observe the filling out in the length/height-for-age chart. Of the 107 CHR, 42.1% had at least one record, regardless of the child's age. There was no report on records of BMI chart for age by the authors of the works included in this review.

Only two studies assessed the presence of records in the development monitoring tool. In Feira de Santana,^23^ 22.1% of CHC had records in the chart, but only 7.8% were complete, considering the child's age. In Belo Horizonte,^25^ only 18.9% of CHR met the criteria for presenting records in three or more age groups.

## Discussion

For three decades, the children health programs in Brazil proposed as a strategy a tool to monitor and promote child health. The results presented in this study have identified important issues in using this instrument to provide the child's primary health care.

Although studies report that most children have the CHC or CHR, the monitoring of child growth seems not to receive the proper attention by health teams. Of the three studies that assessed the CHR,^24^^–^ 26 two presented results regarding the filling out of the HC chart, one regarding the length/height and none regarding BMI for age, regardless of the epidemiological nutritional profile in Brazil. Currently, the coexistence of two antagonistic situations justifies the conduct of different clinical and epidemiological approaches: nutritional deficiency and, at the opposite pole, the combination of problems related to overeating and unhealthy life styles.^39^^,^ 40 As the occurrence of malnutrition declines, the prevalence of anemia, overweight and obesity increases in the Brazilian population.^39^ The IMC has been validated as a marker of adiposity and overweight in children and as a obesity predictor in adulthood.^41^ Therefore, its use is recommended since the child's birth.^42^

To assess the cranial growth rate and its internal structures in childhood, HC systematic measurement and recording on the HC chart for age are needed. It draws attention to a filling out as low as 30.7%^25^ and 35.5%^26^ of a parameter that reflects the state of child neurodevelopment,^43^^–^ 45 so it should be routinely used for individual follow-up of children up to 24 months, the period of greatest postnatal growth.^5^^,^ 45

Low birth weight is one of the best indicators of the quality of health and life of children due to its close relationship with children mortality and damage to the linear growth, weight, and mental and motor development.^46^ However, the low recording of weight at birth in the chart shows the underestimated role assigned to this indicator in monitoring the child's health status at the places evaluated by the works reviewed here.

Another problem found in this review is the poor result in the filling out of the milestones of child development chart. The monitoring action consists of performing physical examination, thorough neuropsychomotor evaluation, identification of risk factors, and record in the CHR of all procedures performed in the child, as well as the findings of the medical visits.^5^ This action is a form of preventive intervention that includes activities related to promotion of normal development and detection of problems in the process.^47^ It brings together different evaluations that include the perception of parents, teachers, and health professionals.^33^^,^ 36 ^,^ 48

An estimated 200 million children worldwide under the age of five are at risk of failing to achieve their development potential.^49^ With the use of CHR, Alvim et al.^36^ were able to trace 35% of children with probable or possible developmental delay, when evaluating 122 children from two months to two years old in the city of Belo Horizonte.

Costa et al.^29^ (2011) found failure in the filling out of CHR when assessing the health care provided to children by the Family Health Program (FHP) in the city of Teixeiras (MG). The authors reported that most children (77.2%) had the CHC, but all (171) were incomplete. There was no information on weight and height, records in the growth chart, and many mothers did not understand the meaning of the curve. The card worked just as a record for vaccine control, and not as a child health monitoring tool.

We also found that the younger children monitoring tools have more records. The schedule for routine medical visits is most common in the first months, a period of risk and need for regular monitoring. Over time, the preventive visits are gradually replaced by visits due to health problems.

The child's health monitoring tool led to operational changes in the health services. Since 2005, hospitals and maternities have become responsible for the distribution and recording of information regarding pregnancy, childbirth, and neonatal period. CHR, as a health promotion tool, also caused changes in health status perceived by the population.^24^ Demand for health services can no longer be motivated only by the presence of disease or vaccination, as reported by Vitolo et al.^50^ in 2010. The findings of this study indicated that 66.2% (n=393) of those responsible still considered the child monitoring by the childcare service unnecessary in the absence of disease. This frequency is in contrast with the high coverage (90%) of the up-to-date immunization schedule.

The results presented in this review should take into account that the methodology used in the articles reviewed to assess the filling out of the CHC and CHR was not uniform. In some studies, the criterion was based on at least one record in the three months preceding the interview. Certainly, the values would be lower than those reported if the criterion used was more restrictive, such as the minimum consultation timetable proposed by the MOH. Another issue to consider is the comparison between surveys performed in different socioeconomic and cultural realities.

Anyway, the absence or records incorrectness suggests a weak link of professionals with basic health care actions and a discontinuity between the actions initiated in maternity and the proposals for primary care.

Health professionals often become overwhelmed in their routines. Beyond the universe of care, the work involves filling out various forms demanded by the institution. The filling out of a CHR cannot be considered an additional administrative record, but a tool for children health promotion and to obtain good quality information to better target the actions of services.

However, it is important to emphasize that the absence of records does not mean exactly the non-performance of medical procedures.^30^^,^ 51 ^,^ 52 However, the importance of records to build the epidemiological profile of a population and as a channel of communication between health professionals in the development of their actions is recognized. When done right, it allows the practice of personalized care and reflects the quality of care.^25^

In the child health monitoring program, the professional focus should be missing no opportunities for action, whether in the promotion and/or prevention and/or assistance, keep bond with the family, and encourage continuous and joint responsibility service and family.^53^ Co-responsibility of families, professionals, and services can be the key to better use the CHR^25^ in child care.

The act of providing explanations, involving the family, and recording information about the child's health conditions is a way of caring for and encouraging the continuity of care. The understanding by the families of this tool function in child health monitoring is essential for them to take hold of it and appreciate it.

## Conclusion

Thirty years after the implementation of the Children Health Integral Assistance Program (PAISC), the use of the child health monitoring tool is not consolidated, according to research reports. The lack of awareness of the health professionals for filling out the study instrument was evident.

This review also shows that the diagnostic of use and filling out quality of such tools in Brazil is restricted to a few local works, which do not evaluate all variables considered essential for child health monitoring. Therefore, further studies are desirable, with a methodology consistent with previous studies that allow drawing a national and more updated picture. This knowledge could be enhanced if combined with other qualitative studies, in which professionals from the basic units and FHP teams express their views on the relationship of promotion and monitoring actions for the child's complete health with the filling out and appreciation of CHR.
