# Supplementary material for: Use of a monitoring tool for growth and development in Brazilian children – systematic review
Source: Rev Paul Pediatr. 2016 Jan-Mar;34(1):122–31. doi: 10.1016/j.rppede.2015.12.002 (PMC4795731; doi:10.1016/j.rppede.2015.12.002)
Supplement: Translation

# Translation: Uso de instrumento de acompanhamento do crescimento e desenvolvimento da criança no Brasil – Revisão sistemática de literatura

**Authors:** Ana Claudia de Almeida, Larissa da Costa Mendes, Izabela Rocha Sad, Eloane Gonçalves Ramos, Vânia Matos Fonseca, Maria Virginia Marques Peixoto

**Affiliations:** a Instituto Nacional de Saúde da Mulher, da Criança e do Adolescente Fernandes Figueira (IFF/Fiocruz), Rio de Janeiro, RJ, Brasil; b Organização Não Governamental Casa da Árvore, Rio de Janeiro, RJ, Brasil

**Keywords:** Saúde da criança, Crescimento e desenvolvimento, Desenvolvimento, Cuidado da criança

## Abstract

**Objetivo:**

Avaliar o uso de instrumento de acompanhamento de saúde da criança, com ênfase nas variáveis do acompanhamento do crescimento e do desenvolvimento, eixo central do cuidado à saúde infantil.

**Fontes dos dados:**

Feita revisão sistemática da literatura de estudos no Brasil nas bases de dados Cochrane Brasil, Lilacs, SciELO e Medline. Os descritores e as palavras-chave usadas foram “crescimento e desenvolvimento”, “desenvolvimento infantil”, “cartão da criança”, “caderneta de saúde da criança”, “cartão e criança” e “caderneta da criança”. Os estudos foram rastreados por título e resumo e foi feita a leitura completa daqueles considerados elegíveis.

**Síntese dos dados:**

Foram identificados 68 artigos e oito foram incluídos no estudo por fazer a análise quantitativa do preenchimento. Cinco estudos avaliaram o preenchimento do Cartão da Criança e três da Caderneta de Saúde da Criança. Todos os artigos concluíram que as informações não foram adequadamente registradas. Os gráficos de acompanhamento do crescimento raramente foram preenchidos e chegaram a 96,3% no caso de peso para a idade. O uso do gráfico do IMC não foi relatado, a despeito do quadro crescente da obesidade infantil. Apenas dois estudos referiram preenchimento dos marcos do desenvolvimento e, nesses, houve registro dos marcos em aproximadamente 20% dos instrumentos verificados.

**Conclusões:**

Os resultados dos artigos revistos evidenciam subutilização do instrumento e refletem baixa sensibilização dos profissionais de saúde para o registro no documento de acompanhamento de saúde da criança.

## Introdução

A função e o uso de um instrumento de acompanhamento da saúde da criança vêm sendo discutidos no âmbito das políticas de atenção básica ao longo das três últimas décadas no país. 1 ^-^ 5 Esse instrumento mudou várias vezes de forma, características e conteúdo. Além disso, teve seus objetivos e seu público alvo ampliados na tentativa de se tornar uma ferramenta efetiva na promoção da saúde da criança. 3 ^,^ 6 ^,^ 7

Nessas mesmas três décadas, as transformações econômicas, sociais e demográficas modificaram o perfil epidemiológico da população brasileira. 8 ^,^ 9 Essas foram acompanhadas por mudanças nas políticas e no sistema de saúde do país,^10^ o que provocou uma reorganização de prioridades na agenda da saúde pública brasileira. 4 ^,^ 5 Muitos avanços foram verificados nos indicadores da atenção básica, como o aumento do acesso aos serviços de pré-natal, de vacinação e das taxas de aleitamento materno. Todos contribuíram para a queda da mortalidade infantil. 8 ^,^ 11 Todas essas transformações apresentaram novos desafios para garantir a saúde do indivíduo em crescimento e desenvolvimento. 12 ^-^ 15 Elas também provocaram a transição de um modelo de atenção centrado nas doenças agudas para um baseado na integração dos serviços de saúde e na promoção intersetorial da saúde. 8 ^,^ 10 ^,^ 16

Nessa transição, desde 1994, o Programa de Saúde da Família (PSF) é a estratégia fundamental de reestruturação do modelo assistencial do Sistema Único de Saúde (SUS).^10^ As equipes de saúde da família constituem o primeiro ponto de contato da população com o sistema de saúde local, coordenam a atenção e procuram integrar os serviços de saúde. As atividades de promoção de saúde ultrapassam os muros das unidades de saúde e passam a ocorrer no território, isto é, nas casas e na comunidade,^10^ e é no exercício dessas atividades que o instrumento de acompanhamento da criança recupera sua função histórica.^17^

As ações feitas na atenção primária à saúde da criança são essenciais para detectar precocemente possíveis alterações de crescimento e desenvolvimento, além de diminuir riscos de morbimortalidade. O crescimento infantil é um processo dinâmico e contínuo de diferenciação desde a concepção até a idade adulta que depende da interação de características biológicas e experiências vivenciadas no meio ambiente. 2 ^,^ 17 O melhor método de acompanhamento desse é o registro periódico de peso, estatura^18^ e, atualmente, do índice de massa corporal (IMC).^5^ O desenvolvimento, por sua vez, é amplo e refere-se a uma transformação progressiva, que inclui, além do crescimento, maturação, aprendizagem e aspectos psíquicos e sociais.^2^ Sua vigilância compreende atividades que avaliam etapas ou marcos do desenvolvimento neuropsicomotor das crianças em cada faixa etária e que podem detectar problemas e alterações no desenvolvimento infantil.^19^

Originalmente, o Cartão da Criança (CC), proposto para o país em 1984,^2^ fazia o monitoramento das ações básicas do Ministério da Saúde (MS) para a saúde infantil. De 1984 a 2003 2 ^,^ 3 o CC foi modificado e revisto e lhe foram acrescentados os direitos da criança e alguns marcos do desenvolvimento infantil. A adoção do CC foi expressamente referida em 2004, na Agenda de Compromissos para a Saúde Integral e Redução da Mortalidade.^4^

Em 2005, o CC assumiu o formato de um livreto e passou a ser denominado Caderneta de Saúde da Criança (CSC). 6 ^,^ 7 Nesse livreto, foram incluídas novas informações destinadas às famílias e aos profissionais de saúde de modo a ampliar o conhecimento do cuidado à criança e a facilitar a compreensão dos aspectos relacionados ao seu crescimento e desenvolvimento. A CSC é considerada pelo MS um instrumento fundamental para monitorar as ações de promoção do pleno potencial de crescimento e desenvolvimento da criança e prevenção dos agravos prevalentes na infância. Atualmente, o MS distribui três milhões de exemplares para as secretarias municipais, que devem repassá-los às maternidades públicas e privadas. É um documento gratuito, entregue à família do recém-nascido. Ainda não foi feito estudo quantitativo que reúna as evidências de estudos anteriores do uso do CC/CSC. 17 ^,^ 20 ^-^ 26 Portanto, o objetivo deste artigo é fazer uma revisão sistemática para avaliar o preenchimento do CC ou da CSC por parte dos profissionais de saúde no Brasil, a partir das evidências publicadas na literatura, com ênfase nas variáveis de acompanhamento do crescimento e do desenvolvimento (CD) da criança.

## Método

A busca foi feita sem restrição ao período de publicação nas bases eletrônicas Cochrane Brasil, Literatura Latino-Americana e do Caribe em Ciências da Saúde (Lilacs), Scientific Electronic Library Online (SciELO), Medical Literature Analysis and Retrieval System Online (Medline) e em listas de referências bibliográficas dos artigos, de acordo com Preferred Reporting Items for Systematic reviews and Meta-Analyses (Prisma).^27^ Os seguintes descritores e palavras-chave foram usados: “crescimento e desenvolvimento”, “desenvolvimento infantil”, “cartão da criança”, “caderneta de saúde da criança”, “cartão e criança” e “caderneta da criança”.

Os artigos incluídos satisfizeram os seguintes critérios relativos à qualidade metodológica:^28^ hipóteses ou objetivos definidos; descrição de desfecho; características dos participantes; variáveis estudadas; principais resultados e características das perdas; adequação dos testes estatísticos usados.

Esta revisão incluiu somente artigos publicados em revistas indexadas feitos no Brasil que mensuravam o uso do instrumento de acompanhamento do crescimento e desenvolvimento elaborado e distribuído pelo Ministério da Saúde, a partir de 1984, e verificou de forma quantitativa o seu preenchimento.

Os critérios de exclusão foram artigos que estavam no formato de resenhas, manuais e trabalhos de conclusão de curso; a metodologia de análise de resultados foi qualitativa; se se detiveram apenas em vacinação ou aqueles cuja amostra era formada por grupos específicos de risco, como baixo peso e prematuridade, portadores de doença genética e com doenças de base.

A versão de 1984 do CC é um folheto em papel cartonado, impresso em cores e formatos diferentes para meninos e meninas, que pode ser dobrado em três, com espaços para anotação de dados de identificação da criança, das consultas, das medidas do peso de acordo com a idade, do gráfico de acompanhamento do crescimento até o quinto ano de idade e do quadro das imunizações feitas. A partir de 1995, o CC incluiu 11 marcos de desenvolvimento infantil com espaços para o registro da idade em que foram alcançados.

A CSC, no formato de livreto que vem sendo reimpresso desde 2005, tem espaços para o registro das informações da atenção básica à saúde da criança da gestação aos nove anos, intercorrências, tratamentos e gráficos para assinalar a variação com a idade de peso, altura, perímetro cefálico (PC) e IMC. Oferece também um quadro para registro da presença dos marcos do desenvolvimento neuropsicomotor de acordo com a idade da criança.

A CSC deve ser preenchida nas consultas de acompanhamento de rotina. O Ministério da Saúde recomenda sete consultas nos primeiros 12 meses (1ª semana e 1°, 2°, 4°, 6°, 9° e 12° mês), duas no segundo ano (18° e 24° mês) e, a partir dessa idade, uma por ano.^7^

## Resultados

Foram identificados 68 artigos não repetidos nas bases eletrônicas de dados e listas de referências bibliográficas (fig. 1). Na primeira etapa de rastreamento foram excluídos, pela leitura de títulos, quatro teses qualitativas e 29 artigos. Desses, 12 estudos restritos à vacinação, nove que envolviam grupos de risco e/ou doença de base, três materiais instrutivos (manuais), três exemplares de cadernetas, uma resenha e um estudo sobre treinamento de profissionais na atenção primária à saúde (APS).

**Figura 1:** Fluxo de identificação, rastreamento, elegibilidade e inclusão dos artigos na revisão sistemática.

Na segunda etapa do rastreamento, 17 artigos foram excluídos após a leitura de resumos por não verificarem o preenchimento do CC/CSC. Onze artigos podem ser agrupados como estudos de avaliação: três de indicadores nutricionais, três de APS, dois de práticas do cuidado, dois de análise de prontuário e um de conhecimento de profissionais. Cinco artigos podem ser agrupados como estudo de metodologia qualitativa: dois estudos de significado do cuidado da criança, um de análise de discurso, um relato de experiência e uma abordagem multidisciplinar do acompanhamento do CD. Além desses, uma revisão bibliográfica sobre a atuação do enfermeiro em saúde nutricional da criança.

Dezoito artigos permaneceram para leitura completa do texto. Dez 29 ^-^ 38 foram excluídos por não fazerem avaliação quantitativa do preenchimento dos instrumentos de acompanhamento de saúde da criança (tabela 1). Dos oito artigos incluídos (tabela 2), cinco avaliaram o preenchimento do CC 17 ^,^ 20 ^-^ 23 e três da CSC. 24 ^-^ 26 As pesquisas foram feitas nas regiões Nordeste, 17 ^,^ 21 ^,^ 23 Sudeste, 20 ^,^ 24 ^,^ 25 Sul^26^ e Centro Oeste.^22^

**Tabela 1:** Trabalhos selecionados para leitura integral e excluídos da revisão sistemática

Título	Autor (es)	Causa da exclusão
Avaliação da atenção à saúde da criança no contexto da Saúde da Família no município de Teixeiras, Minas Gerais (MG, Brasil).	Da Costa *et al* . 29	A classificação utilizada para análise do preenchimento do CC foi “incompleta” sem especificação de frequências para as variáveis de interesse.
O Programa de Agentes Comunitários de Saúde no Ceará: o caso de Uruburetama.	Ávila 30	As ações de acompanhamento do crescimento e do desenvolvimento infantil foram avaliadas quanto aos aspectos técnicos e práticas dos agentes.
Avaliação nutricional de crianças de seis a sessenta meses.	Sousa & Araújo 31	A avaliação nutricional das crianças foi realizada por comparação entre os critérios de *Waterlow* e da curva peso/idade do CC.
Evolução da assistência materno‐infantil e do peso ao nascer no Estado de Pernambuco em 1997 e 2006.	Noronha *et al* . 32	As informações foram coletadas com o responsável da criança. O peso ao nascer foi coletado do CC quando registrado.
Avaliação da atenção à saúde da criança (0‐5 anos) no PSF de Teresópolis (RJ) segundo a percepção dos usuários.	Ribeiro *et al* . 33	A vinculação com instituições públicas de atenção e cuidado infantil foi evidenciada pela posse do CC.
Vigilância do crescimento infantil: conhecimento e práticas de enfermeiros da atenção primária à saúde.	Reichert *et al* . 34	Os gráficos de crescimento foram utilizados em questões de verificação do conhecimento de profissionais.
Desenvolvimento infantil: concordância entre a caderneta de saúde da criança e o manual para vigilância do desenvolvimento infantil.	Oliveira *et al* . 35	Foram comparadas as classificações do desenvolvimento segundo a CSC e o Manual para Vigilância do Desenvolvimento Infantil no Contexto da AIDPI.
A avaliação do desenvolvimento infantil: um desafio interdisciplinar.	Alvim *et al* . 36	Foram comparadas as classificações do desenvolvimento segundo a CSC e o Manual para Vigilância do Desenvolvimento Infantil no Contexto da AIDPI.
Características neuromotoras, pôndero‐estaturais e biopsicossociais de lactentes.	Rothstein & Beltrame 37	O Cartão de Saúde da criança foi utilizado apenas como documento fonte de informações.
Acolhimento e características maternas associados à oferta de líquidos a lactentes.	Niquini *et al* . 38	As mães foram questionadas se receberam o cartão de acolhimento mãe–bebê na maternidade. Não houve análise do preenchimento de variáveis.

**Tabela 2:** Descrição dos trabalhos incluídos na revisão sistemática

Autores	Objetivo	Local coleta dados	Conclusão
Santos *et al* . 20	Analisar os cuidados primários prestados à população materno‐infantil.	Posto de vacinação da cidade Teresópolis (RJ).	Mesmo a consulta sendo de puericultura, 30% das crianças não tinham seu peso registrado no CC.
Ratis & Batista 17	Avaliar estrutura e processo da vigilância do crescimento.	Unidades de saúde do Estado de PE.	O desinteresse pela monitoração do crescimento foi mais proeminente no interior.
Carvalho *et al* . 21	Analisar o acompanhamento do crescimento.	Unidades de saúde do Estado de PE.	Os indicadores da monitoração do crescimento não foram muito além de 50%, sendo menores no interior.
Sardinha & Pereira 22	Verificar o preenchimento do CC.	Centros de saúde nas cidades do DF.	O preenchimento do gráfico de peso foi mais realizado nas crianças de menor idade.
Vieira *et al* . 23	Verificar o preenchimento do CC.	Unidades de saúde de Feira de Santana (BA).	O preenchimento da curva de crescimento estava completo em 41,1% (905) e do quadro de desenvolvimento em 7,8% (170).
Goulart *et al.* 24	Avaliar o preenchimento e conhecer a percepção das mães sobre a CSC.	UBS de Belo Horizonte (MG) e visita domiciliar.	O peso ao nascer foi o campo mais preenchido (91%). As falhas sugerem que a CSC não cumpre seu objetivo.
Alves *et al* . 25	Analisar a qualidade do preenchimento da CSC.	UBS de Belo Horizonte (MG).	Os melhores percentuais de preenchimento foram relativos à identificação, ao registro de vacinas e aos dados ao nascer.
Linhares *et al* . 26	Avaliar o preenchimento e conhecer a percepção das mães sobre a CSC.	Visita domiciliar em áreas de quatro UBS de Pelotas (RS).	O preenchimento da CSC estava limitado às seções que já constavam do CC.

As informações foram obtidas de questionários dirigidos à mãe ou ao responsável pela criança, ou aos diretores de unidades, ou foram coletadas diretamente do instrumento estudado. Os inquéritos foram feitos em serviços pertencentes à rede pública e em visitas domiciliares.

A variabilidade de itens mensurados e de critérios de avaliação do preenchimento dificultou a comparação da frequência de preenchimento para todos os itens do CC ou da CSC.

O percentual de preenchimento dos dados de identificação, acompanhamento da gestação e nascimento é apresentado na tabela 3. Em 2005, apenas 55,6% das CSC tinham o nome da criança preenchido.^24^ Os autores relataram que a média de idade dessas “crianças sem nome” foi de 68 dias (2,2 meses), mediana de 59 dias (1,9 mês), época em que essa informação já deveria ter sido preenchida pelos profissionais de saúde após diversas oportunidades de contato com a criança: na maternidade e em atendimentos e consultas na atenção básica. Observa-se também que houve aumento do percentual de preenchimento, entre 2005 e 2008, para todas as variáveis de identificação, exceto para o número da Declaração de Nascido Vivo (DNV). O maior aumento foi verificado no número de Registro Civil de Nascimento (RCN): quatro vezes mais.

**Tabela 3:** Percentual de preenchimento dos dados de identificação, acompanhamento da gestação e nascimento relatados nos estudos incluídos na revisão sistemática a

Autores		Ratis & Batista F. 17	Carvalho *et al.* 21	Vieira *et al* . 23	Goulart *et al* . 24	Alves *et al* . 25	Linhares *et al* . 26
*Ano pesquisa*		1998	1998	2001	2005	2006	2008
*Documento*		CC	CC	CC	CSC	CSC	CSC
*N*		1194	662	2215	797	355	107
*Idade*		<5 anos	<12 meses	≤12 meses	<9 meses	<16 meses	<12 meses

*Identificação*							
	Nome	–	–	99,8	55,6	93,8	93,5
	Data nasc.	–	–	99,3	90,1	99,7	100
	Local nasc.	–	–	76,6	–	–	98,1
	Nome mãe	–	–	–	90,7	98,9	99,1
	Endereço	–	–	–	38,9	–	73,8
	Telefone	–	–	–	22,1	–	47,7
	Bairro	–	–	–	33,4	–	67,3
	CEP	–	–	–	14,6	–	21,5
	Cidade	–	–	–	34,3	–	64,5
	Etnia/Cor	–	–	–	50,1	–	66,4
	N° DNV	–	–	–	60,9	–	33,6
	N° RCN	–	–	–	2,0	–	8,4

*Gestação*							
	Pré‐natal	–	–	–	59,6	58,0	–
	N° consulta pré‐natal	–	–	–	68,5	69,9	–
	Sorologia	–	–	–	50,0	–	–
	Tipo parto	–	–	93,3	84,9	89,3	–
	Nascimento						
	Idade gestacional	–	–	–	75,8	72,4	–
	Apgar5’	–	–	28,4	76,7	53,5	–
	Peso	86,8	89,4	97,2	91,1	96,9	–
	Comprimento	–	–	91,8	89,6	91,2	–
	Perímetro cefálico	–	–	88,9	84,9	85,6	–

a Os estudos de Santos *et al.* 20 e de Sardinha & Pereira 22 não apresentaram resultado de preenchimento de identificação, acompanhamento da gestação e nascimento.

DNV, Declaração de Nascido Vivo; RCN, Registro Civil de Nascimento.

Apenas um estudo avaliou o preenchimento dos dados de sorologia no pré-natal^24^ e verificou que essa foi a menor porcentagem de preenchimento das variáveis de acompanhamento da gestação: aproximadamente 50% das CSC estudadas.

O registro do peso ao nascer foi o mais descrito dentre as variáveis relativas ao nascimento da criança (tabela 3). As porcentagens de preenchimento foram crescentes entre os CC estudados, mas houve queda quando na mudança do instrumento, como, por exemplo, na idade gestacional. Entre 2001 e 2006, houve aumento no preenchimento do Apgar e pouca variação no preenchimento de comprimento e perímetro cefálico.

Os resultados das variáveis de acompanhamento do crescimento e desenvolvimento foram reunidos na tabela 4. Apenas dois trabalhos 20 ^,^ 26 relataram registros de consultas relativos ao crescimento. O menor percentual de preenchimento foi 74,6%, acompanhamento de peso, em 1998.^20^ Porém, dez anos depois, os registros de peso, estatura e PC obtiveram mais de 80% de preenchimento no trabalho de Linhares *et al*.^26^

**Tabela 4:** Percentual de preenchimento das variáveis de acompanhamento do crescimento e desenvolvimento nos estudos incluídos na revisão sistemática *

Autores		Santos *et al* . 20	Ratis & Batista F. 17	Carvalho *et al.* 21	Sardinha & Pereira 22	Vieira *et al* . 23	Alves *et al* . 25	Linhares *et al* . 26
*Instrumento*		CC	CC	CC	CC	CC	CSC	CSC
*Idade*		<12 meses	<5 anos	<12 meses	<5 anos	≤12 meses	<16 meses	<12 meses
*Crescimento*								
*Dados das consultas*								
		( *N* =299)						( *N* =107)
	Data da consulta							91,6
	Idade							90,7
	Peso	74,6 a						89,7 b
	Estatura							87,9 b
	Perímetro cefálico							82,2 b

*Dados de nascimento nos gráficos*								
			( *N* =1193)	( *N* =662)			( *N* =355)	
	Peso		36,9	44,1			69,3	
	Perímetro cefálico						15,5	

*Dados das consultas nos gráficos*								
		( *N* =307)	( *N* =624)	( *N* =402)	( *N* =3543)	( *N* =2200)	( *N* =355)	( *N* =107)
	Peso	70,4 a	59,9 c	58,2 d	21,1 e	41,1 f	59,4 g	96,3 h
	Comprimento/altura							42,1 h
	Perímetro cefálico						30,7 g	35,5 h
	Desenvolvimento							
						( *N* =2191)	( *N* =355)	
	Marcos de 0 a 36 meses					7,8 i	18,9 j	

* Goulart *et al* . 24 não apresentaram dados de preenchimento das variáveis de acompanhamento do crescimento e desenvolvimento.

a Pelo menos um registro nos três meses que antecederam à entrevista.

b Registros de acordo com a idade da criança.

c Registros no dia da consulta.

d Último registro atualizado.

e Registros corretamente pontuados, segundo o Ministério da Saúde.

f Pelo menos um registro a cada três meses.

g Registros de peso e de PC marcados no gráfico cuja diferença entre a idade na data do registro e a idade cronológica da criança foi ≤ 3 meses.

h Pelo menos um registro verificado.

i Todos os registros correspondentes à idade da criança.

j Registros em três ou mais faixas etárias disponíveis na CSC.

Os registros de peso e PC ao nascer nos gráficos apresentaram baixa frequência de preenchimento. Nos trabalhos feitos em Pernambuco, o peso ao nascer só foi assinalado no gráfico em 36,9%^17^ e 44,1%^21^ dos cartões, embora estivesse registrado em 86,8%^17^ e 89,4%^21^ (tabela 4), respectivamente, desses cartões. Da mesma forma, em Belo Horizonte,^25^ apenas 69,3% e 15,5% das CSC continham as marcações nos gráficos de peso e de PC ao nascer, respectivamente.

O percentual de preenchimento do gráfico de peso para idade apresentou grande variação entre os estudos (21,1% a 96,3%) devido aos critérios usados para considerar o preenchimento como adequado. Para crianças com até um ano, quando foi exigido um registro a cada três meses, Vieira *et al*.^23^ relataram 41,1% de preenchimento adequado no gráfico de peso para idade. No estudo que considerou suficiente apenas uma marcação, foi relatado um percentual de preenchimento de 96,3%.^26^ No Distrito Federal,^22^ foram encontrados 21,1% de preenchimento correto, de acordo com o recomendado pelo Ministério da Saúde. Foi observado que o percentual de preenchimento diminuiu com a idade, caiu de 53,8% na faixa até cinco meses para 6,6% na faixa de 48 a 60 meses. Em Pernambuco,^17^ 59,9% dos CC apresentavam registro no gráfico de peso no dia da consulta. Nesse mesmo trabalho, de acordo com a idade da criança, 38% dos CC apresentavam nenhum ou apenas um registro de peso no gráfico. A condição “nenhum ponto registrado” distribuiu-se de forma semelhante em todos os grupos etários: 27,8% (<12 meses), 21,7% (12-24 meses) e 27,2% (48-60 meses). Porém, 40,5% dos CC apresentavam de dois a seis pontos no gráfico. Desses, 46% no grupo menor de um ano e 29,7% entre 48-60 meses.

Linhares *et al*.^26^ foram os únicos a observar preenchimento no gráfico de comprimento/altura por idade. Das 107 CSC, 42,1% apresentaram pelo menos um registro, independentemente da idade da criança. Não foram mencionados registros no gráfico de IMC para idade pelos autores dos trabalhos incluídos nesta revisão.

Apenas dois trabalhos avaliaram a presença de registros no instrumento de vigilância do desenvolvimento. Em Feira de Santana,^23^ 22,1% dos CC apresentaram registros no quadro, mas apenas 7,8% estavam completos, considerando-se a idade da criança. Em Belo Horizonte,^25^ somente 18,9% das CSC satisfizeram o critério de apresentar registros em três ou mais faixas etárias.

## Discussão

Há três décadas os programas de saúde da criança no Brasil propõem como estratégia um instrumento para monitorar e promover a saúde infantil. Os resultados apresentados no presente estudo permitiram identificar questões importantes no uso desse instrumento para prestar cuidados primários de saúde da criança.

Embora os trabalhos relatem que a maioria das crianças tem o CC ou a CSC, a monitoração do crescimento infantil parece ainda não receber a devida atenção pelas equipes de saúde. Dos três trabalhos que avaliaram a CSC, 24 ^-^ 26 dois apresentaram resultados quanto ao preenchimento do gráfico de PC, um de comprimento/altura e nenhum de IMC para idade, a despeito do perfil epidemiológico nutricional no Brasil. Atualmente, a coexistência de duas situações antagônicas justifica a conduta de enfoques clínicos e epidemiológicos diferenciados: deficiência nutricional e, no polo oposto, a associação de problemas relacionados aos excessos alimentares e estilos de vida não saudáveis. 39 ^,^ 40 À medida que declina a ocorrência da desnutrição, aumenta a prevalência de anemia, sobrepeso e obesidade na população brasileira.^39^ O IMC já foi validado em crianças como marcador de adiposidade e sobrepeso e preditor de obesidade na idade adulta.^41^ Por isso, recomenda-se seu uso desde o nascimento da criança.^42^

Na infância, para verificar a velocidade de crescimento craniano e suas estruturas internas, é necessária a mensuração sistemática do PC e o registro no gráfico de PC para idade. Chama atenção tão baixo preenchimento (30,7%^25^ e 35,5%^26^) de um parâmetro que reflete o estado do neurodesenvolvimento infantil 43 ^-^ 45 e, por isso, deve ser rotineiramente usado para seguimento individual de crianças até 24 meses, período de maior crescimento pós-natal. 5 ^,^ 45

O baixo peso ao nascer é um dos melhores indicadores da qualidade de saúde e de vida das crianças devido à sua estreita relação com a mortalidade infantil e com os prejuízos para o crescimento linear, ponderal e desenvolvimento mental e motor.^46^ Contudo, o baixo registro de peso ao nascer no gráfico evidencia o subestimado papel atribuído a esse indicador no acompanhamento do estado de saúde da criança nos locais avaliados pelos trabalhos aqui revisados.

Outro problema evidenciado nesta revisão é o baixo resultado no preenchimento do quadro de acompanhamento dos marcos de desenvolvimento da criança. A ação de vigilância consiste em fazer sistematicamente exame físico, avaliação neuropsicomotora de maneira minuciosa, identificação da presença de fatores de risco e registro, na CSC, de todos os procedimentos feitos na criança, bem como dos achados das consultas.^5^ Essa ação constitui uma modalidade de intervenção preventiva que compreende atividades relacionadas à promoção do desenvolvimento normal e à detecção de problemas nesse processo.^47^ Reúne diferentes avaliações que incluem a percepção dos pais, professores e profissionais de saúde. 33 ^,^ 36 ^,^ 48

Estima-se que, no mundo, 200 milhões de crianças com menos de cinco anos estão em risco de não atingir plenamente o seu potencial de desenvolvimento.^49^ Pelo uso da CSC, Alvim *et al*.^36^ conseguiram rastrear 35% de crianças com provável ou possível atraso de desenvolvimento, ao avaliar 122 crianças de dois meses a dois anos de idade, em Belo Horizonte.

Falhas no preenchimento são reiteradas por Costa *et al*. (2011)^29^ ao verificarem a atenção dada à saúde da criança pelo Programa de Saúde da Família (PSF) em Teixeiras (MG). Os autores relataram que a maioria das crianças (77,2%) tinha o cartão da criança, mas todos (171) estavam incompletos. Não tinham informações sobre o peso e altura, registros no gráfico de crescimento e muitas mães não compreendiam o significado da curva. O cartão funcionava apenas como um registro para o controle de vacina, e não como um instrumento de acompanhamento de saúde da criança.

Observou-se que os registros são mais efetuados nos instrumentos de crianças de menor idade. O agendamento para as consultas de rotina é mais frequente nos primeiros meses, época de risco e com maior necessidade de acompanhamento periódico. Com o passar do tempo, as consultas preventivas são gradativamente substituídas por consultas por agravos à saúde.

A CSC provocou mudanças operacionais nos serviços de saúde. A partir de 2005, hospitais e maternidades tornaram-se responsáveis pela distribuição e registro das informações relativas a gravidez, parto e período neonatal. A CSC, como instrumento de promoção de saúde, provocou alterações também na percepção de estado de saúde pela população.^24^ A demanda por serviços de saúde não pode mais ser motivada apenas pela presença de doença ou por vacinação, como observado por Vitolo *et al*.,^50^ em 2010. As conclusões desse estudo indicaram que 66,2% (n=393) dos responsáveis ainda consideravam desnecessário o acompanhamento da criança pelo serviço de puericultura na ausência de doença. Essa frequência contrastou com a elevada cobertura (90%) do calendário de imunização em dia.

Os resultados apresentados nesta revisão devem levar em consideração que a metodologia usada nos artigos revistos para verificação do preenchimento dos CC e das CSC não foi uniforme. Em alguns estudos, o critério baseou-se em pelo menos um registro nos três meses que antecederam a entrevista. Certamente, os valores obtidos seriam menores do que os relatados se o critério usado fosse mais restritivo, como o calendário mínimo de consultas, proposto pelo MS. Outra questão a ser considerada é a comparação entre pesquisas feitas em diferentes realidades socioeconômicas e culturais.

De qualquer forma, a ausência ou incorreção de registros sugere um fraco vínculo dos profissionais com as ações básicas de saúde e descontinuidade entre as ações iniciadas na maternidade e as propostas para a atenção básica.

Profissionais de saúde, muitas vezes, ficam sobrecarregados em suas rotinas. Além do universo da assistência, o trabalho envolve o preenchimento de vários formulários demandados pela instituição. O preenchimento da CSC não pode ser considerado mais um registro administrativo, mas uma ferramenta de promoção de saúde da criança e de obtenção de informação de boa qualidade, para melhor direcionar as ações dos serviços.

Entretanto, é importante ressaltar que a ausência de registro não significa exatamente a não feitura de procedimentos. 30 ^,^ 51 ^,^ 52 No entanto, é reconhecida a importância dos registros para a construção do perfil epidemiológico de uma população e como um canal de comunicação entre os profissionais de saúde no desenvolvimento de suas ações. Quando bem feito, permite a prática do cuidado personalizado e reflete a qualidade da assistência prestada.^25^

No programa de vigilância da saúde da criança, o foco do profissional deveria ser o de não perder oportunidades de atuação, seja na promoção e/ou na prevenção e/ou na assistência, manter o vínculo com a família e estimular a responsabilidade contínua e conjunta serviço e família.^53^ A corresponsabilização de famílias, profissionais e serviços pode ser a chave para melhor uso da CSC^25^ no cuidado à criança.

O ato de fornecer explicações, envolver a família e registrar as informações sobre as condições de saúde da criança são formas de cuidar e de estimular a continuidade do cuidado. A compreensão pela família da função desse instrumento no acompanhamento de saúde infantil é fundamental para que ela dele se aproprie e o valorize.

## Conclusão

Trinta anos após a implantação do Programa de Assistência Integral à Saúde da Criança (PAISC), o uso do instrumento de vigilância da saúde da criança não está consolidado, segundo os relatos das pesquisas feitas. Ficou evidente a falta de sensibilização dos profissionais das equipes de saúde para o preenchimento do instrumento estudado.

Esta revisão mostra também que o diagnóstico de uso e qualidade de preenchimento de tais instrumentos no Brasil está restrito a poucos trabalhos locais, que não avaliam todas as variáveis consideradas imprescindíveis para a vigilância da saúde da criança. Portanto, são desejáveis mais estudos dessa natureza, com metodologia compatível com os estudos anteriores, que possibilitem traçar um panorama nacional e mais atualizado. Esse conhecimento poderia ser enriquecido se associado a outros estudos de natureza qualitativa, nos quais os profissionais das unidades básicas e equipes do PSF expressassem suas opiniões sobre a relação das ações de promoção e de vigilância da saúde integral da criança com o preenchimento e a valorização da CSC.
